# A revised nomenclature for transcribed human endogenous retroviral loci

**DOI:** 10.1186/1759-8753-2-7

**Published:** 2011-05-04

**Authors:** Jens Mayer, Jonas Blomberg, Ruth L Seal

**Affiliations:** 1 Department of Human Genetics, Theoretical Medicine and Biosciences, Centre for Human and Molecular Biology, Saarland University, D-66421 Homburg, Germany; 2 Section of Virology, Department of Medical Sciences, Uppsala University, SE-751 85 Uppsala, Sweden; 3 HUGO Gene Nomenclature Committee, European Molecular Biology Laboratory-European Bioinformatics Institute, Wellcome Trust Genome Campus, Hinxton, Cambridgeshire CB10 1SD, UK

## Abstract

**Background:**

Endogenous retroviruses (ERVs) and ERV-like sequences comprise 8% of the human genome. A hitherto unknown proportion of ERV loci are transcribed and thus contribute to the human transcriptome. A small proportion of these loci encode functional proteins. As the role of ERVs in normal and diseased biological processes is not yet established, transcribed ERV loci are of particular interest. As more transcribed ERV loci are likely to be identified in the near future, the development of a systematic nomenclature is important to ensure that all information on each locus can be easily retrieved.

**Results:**

Here we present a revised nomenclature of transcribed human endogenous retroviral loci that sorts loci into groups based on Repbase classifications. Each symbol is of the format ERV + group symbol + unique number. Group symbols are based on a mixture of Repbase designations and well-supported symbols used in the literature. The presented guidelines will allow newly identified loci to be easily incorporated into the scheme.

**Conclusions:**

The naming system will be employed by the HUGO Gene Nomenclature Committee for naming transcribed human ERV loci. We hope that the system will contribute to clarifying a certain aspect of a sometimes confusing nomenclature for human endogenous retroviruses. The presented system may also be employed for naming transcribed loci of human non-ERV repeat loci.

## Human endogenous retroviruses

Human endogenous retroviruses (ERVs) are remnants of infections of former exogenous retroviruses. Proviruses formed by numerous distinct exogenous retroviruses in the germline genome could be inherited by subsequent generations. About 8% of the human genome consists of sequences that are potentially of retroviral origin [1] and are distributed in about 700,000 different loci. In addition to proviruses, these sequences include solitary long terminal repeats (LTRs), nonretroviral sequences flanked by LTRs that may not be directly derived from infectious retroviruses and sequences similar to LTRs. ERVs and related sequences are thus part of the repetitive portions of the human genome, which comprise about 45% of the human genome mass, including mobile DNA such as L1, Alu and SVA elements.

Detailed analysis of the human genome sequence by wet-lab and bioinformatics approaches resulted in the definition of ERV groups, with the number depending on the methods used for defining groups: 31 groups were defined by Sperber *et al. *[2] and Blomberg *et al. *[3], 42 groups were defined by Mager and Medstrand [4], 30 groups were defined by Gifford and Tristem [5] and several hundred human ERV and LTR families were defined by Repbase [6].

Almost all human ERV loci no longer encode former retroviral proteins because of their ancient incorporation into the host genome and thus accumulation of nonsense mutations. Many loci are missing large proviral portions, and most loci have been reduced to so-called solitary LTRs by homologous recombination between proviral LTRs. For more detailed information on human ERVs, we refer interested readers to recent reviews on the topic and the references therein [7-10].

While protein coding capacity is very limited, many human ERV loci still are transcribed and usually are initiated by promoter sequences within the proviral LTRs. Obviously, mutations within LTRs have not yet rendered all LTRs in the human genome defective. In principle, promoters in flanking, non-ERV sequences may also contribute to transcription of those loci. Probably every human tissue and cell type, diseased or not, contains ERV transcripts [11,12]. More than a single ERV group is usually found transcribed, and patterns of transcribed ERV groups differ between tissue and cell types. Transcription of ERV loci is thus regulated in some way. While expression of ERV sequences has been associated with a number of human diseases, such as germ cell tumours, melanoma and multiple sclerosis, the involvement of ERVs in human diseases remains to be elucidated. On the other side, some ERV loci very likely provide important biological functions, such as the syncytin [13] and syncytin 2 loci [14], referred to herein as ERVW-1 and ERVFRD-1, respectively. Other loci harbouring only partial open reading frames, such as a recently characterized HERV-W locus on chromosome Xq22.3 [15] (ERVW-2), may likewise produce partial retroviral proteins with potential biological functions. It is therefore of particular interest which ERV loci actually contribute to the human transcriptome.

Recent studies have identified transcribed ERV loci in normal and diseased human cells and tissues by means of reassigning ERV cDNA sequences to individual loci in the human reference genome sequence, employing characteristic nucleotide differences between individual loci of a regarded ERV group. Many more transcribed ERV loci are likely to be identified in future studies. It is therefore necessary to introduce a nomenclature for transcribed human ERV sequences.

## Previous nomenclature used in the literature

The lack of an established nomenclature for transcribed ERV elements has led to confusion within the literature. These problems were previously reviewed in detail [16]. ERVs have been classified into groups (formerly known as "families", which is heresy to virologists because "family" refers to *Retroviridae*), although different classification systems have been used. For instance, some groups have been defined initially by molecular genetics means, others by sequence similarity and others by primer binding site sequences. Changing amounts of sequence information also showed that some ERV groups' designations needed to be revised. Different names have been used for the same ERV group. Likewise, individual loci have been referred to using a variety of different symbols (for example, see the aliases listed in Table 1 for the ERVK-6 locus). The use of different symbols for the same locus makes it difficult to retrieve all information on that particular locus.

**Table 1:** Nomenclature for transcribed human endogenous retrovirus loci

Symbol	Name	Chromosome location	Aliases	Representative GenBank accession numbers	Sources
ERVK-1^a^	endogenous retrovirus group K, member 1	1p31.1	c1_A	FN806826, BQ304053	[23] [24]

ERVK-2	endogenous retrovirus group K, member 2	3p25.3	c3_A	FN806829, EF153331	[23]

ERVK-3	endogenous retrovirus group K, member 3	3q13.2	c3_B	FN806830	[23]

ERVK-4	endogenous retrovirus group K, member 4	3q21.2	c3_C, ERVK4, HERV-K(I)	FN806831	[23] [25]

ERVK-5	endogenous retrovirus group K, member 5	3q12.3	ERVK5, HERV-K(II)	CF227259, AK021866	[24] [25]

ERVK-6	endogenous retrovirus group K, member 6	7p22.1	c7_A, ERVK6, HERV-K108, HERV-K(HML-2.HOM), envK2, HERV-K(C7)	FN806837, AY371029, X82271, AF080233	[23] [26] [27]

ERVK-7	endogenous retrovirus group K, member 7	1q22	c1_B, ERVK7, HERV-K102	FN806827, EF153338, S46404, DQ069911	[23] [28]

ERVK-8	endogenous retrovirus group K, member 8	8p23.1	c8_A, ERVK8, HERV-K115, envK6	FN806840	[23] [27] [29]

ERVK-9	endogenous retrovirus group K, member 9	6q14.1	c6_A, HERV-K109, envK4	FN806836, AF080234, AY371030	[23] [26] [27] [28]

ERVK-10	endogenous retrovirus group K, member 10	5q33.3	c5_A, HERV-K10	FN806835, AF080231, CN345079	[23] [24] [26]

ERVK-11	endogenous retrovirus group K, member 11	3q27.2	c3_E, N8.4, HML-2A	FN806833, AF080232, AF080229, U87590	[23,26]

ERVK-12	endogenous retrovirus group K, member 12	4q32.3	c4_A	FN806834, EF153341	[23]

ERVK-13	endogenous retrovirus group K, member 13	3q24	c3_D	FN806832	[23]

ERVK-14	endogenous retrovirus group K, member 14	7q22.1	c7_B	FN806838	[23]

ERVK-15	endogenous retrovirus group K, member 15	7q34	c7_C, P1.10	FN806839, U87594	[23]

ERVK-16	endogenous retrovirus group K, member 16	10p14	c10_A. M3.8	FN806841, EF543114, U87587	[23] [27] [30]

ERVK-17	endogenous retrovirus group K, member 17	10q24.2	c10_B	FN806842, AF080230	[23] [26]

ERVK-18	endogenous retrovirus group K, member 18	1q23.3	c1_C, HERV-K18	FN806828	[23] [31]

ERVK-19	endogenous retrovirus group K, member 19	19q11	P1.8, HERV-K(C19), envK3	U87593	[27,30] [31]

ERVK-20	endogenous retrovirus group K, member 20	11q23.3	c11_B	FN806844	[23]

ERVK-21	endogenous retrovirus group K, member 21	12q14.1	c12_A, envK1	FN806845, U32496	[23] [27]

ERVK-22	endogenous retrovirus group K, member 22	19p13.3	c19_A	FN806846, EF153351	[23]

ERVK-23	endogenous retrovirus group K, member 23	21q21.1	c21_A	FN806847, EF543113	[23]

ERVK-24	endogenous retrovirus group K, member 24	22q11.21	c22_A, HERV-K101	FN806848, AU124350, AA580921, AW812040	[23] [28]

ERVK-25	endogenous retrovirus group K, member 25	11q22.1	c11_A	FN806843, CF227268, AW818206	[23]

ERVK3-1^b^	endogenous retrovirus group K3, member 1	19q13.43	HERV-K(HML6-1)	AK054868, BC010118, BC011670	[32]

ERVK3-2	endogenous retrovirus group K3, member 2	14q24.2	HML6-c14	AK027828, AK096726, CR591084	[32]

ERVK3-3	endogenous retrovirus group K3, member 3	5q13.2	HML6-c5	FR714893	[33]

ERVK3-4	endogenous retrovirus group K3, member 4	11p15.4	HML6-c11	FR714894	[33]

ERVK3-5	endogenous retrovirus group K3, member 5	12q24.12	HML6-c12	FR714895	[33]

ERVK3-6	endogenous retrovirus group K3, member 6	19q13.41	HML6-c19A	FR714896	[33]

ERVK3-7	endogenous retrovirus group K3, member 7	19p13.2	HML6-c19B	FR714897	[33]

ERVK3-8	endogenous retrovirus group K3, member 8	20p11.21	HML6-c20	FR714898	[33]

ERVFRD-1	endogenous retrovirus group FRD, member 1	6p24.2	HERV-FRD, envFRD, ERVFRDE1, syncytin 2	AK075092, AK123938, AY358244	[24] [27] [34]

ERVFRD-2^c^	endogenous retrovirus group FRD, member 2	19q13.41	FLJ45949	AK127846	

ERV3-1	endogenous retrovirus group 3, member 1	7q11.21	ERV3, envR	AK295189	[24] [27] [35] [36]

ERV3-2^c^	endogenous retrovirus group 3, member 2	7q33	KIAA1466	AB040899, AL833192	

ERVPABLB-1	endogenous retrovirus group PABLB, member 1	3p24.3	envR(b)	BQ012865, CF529244, AI189490	[27]

ERVFC1-1	endogenous retrovirus group FC1, member 1	7q36.2	envF(c)2	AK124285	[27]

ERVW-1	endogenous retrovirus group W, member 1	7q21.2	ERVWE1, syncytin 1, enverin, envW, HERV-W-ENV, HERV-7q, HERV7Q	BG012022, AF208161, BX391741, BX365066	[13,24] [27] [37]

ERVW-2	endogenous retrovirus group W, member 2	Xq22.3	ERVWE2, Penv-C15	AF127228, FN689795	[38]

ERVW-3	endogenous retrovirus group W, member 3	3q23	CL4	AF127227	[39]

ERVW-4	endogenous retrovirus group W, member 4	15q21.3	C187-23	AF123882, EF539878	[38] [39] [40]

ERVW-5	endogenous retrovirus group W, member 5	3q26.32	CL2	AF123881	[39]

ERVW-6	endogenous retrovirus group W, member 6	11q14.1		AK022306, AB063619	[41]

ERVS71-1	endogenous retrovirus group S71, member 1	19p13.11	envT	CN288807, BQ932595, BQ941761	[27] [42]

ERVS71-2	endogenous retrovirus group S71, member 2	10p11.1	HERV-HC2	AB167270, AB167277	[43]

ERVFH21-1	endogenous retrovirus group FH21, member 1	7p21.3	HERV-F(XA34)	AK023847	[32]

ERVH48-1	endogenous retrovirus group 48, member 1	21q22.3	C21orf105, HERV-F (type b)	BC005107, CR591419	[32]

ERVE-1	endogenous retrovirus group E, member 1	17q11.2	ERVE1	BC037342, FM212572	[44]

ERVE-2	endogenous retrovirus group E, member 2	11q13.2		FM212575	[45]

ERVE-3	endogenous retrovirus group E, member 3	8p23.1		FM212573	[45]

ERVE-4	endogenous retrovirus group E, member 4	6q15	CT-RCC-1	EU137846	[46]

ERVV-1	endogenous retrovirus group V, member 1	19q13.41	HERV-V1, ENVV1	AK056776, BC104018, BC104019	[47]

ERVV-2	endogenous retrovirus group V, member 2	19q13.41	HERV-V2, ENVV2	AI434519, CA417098, DA863698	[47]

ERVI-1^c^	endogenous retrovirus group I, member 1	9q22.1		AK124340, AK124077, CR614956	

ERV18-1^c^	endogenous retrovirus group 18, member 1	15q21.3		AK126787	

ERVMER61-1^c^	endogenous retrovirus group MER61, member 1	1q31.3	C1orf99	BC040856	

ERVH-1	endogenous retrovirus group H, member 1	4p15.2	HERV-H4p15.2	EU669866, BC015108	[48]

ERVH-2	endogenous retrovirus group H, member 2	Xp22.32	HERV-HX	EU195218, EU195219	[48]

ERVH-3	endogenous retrovirus group H, member 3	6q12	HERV-H/F	AJ431196	[49]

ERVH-4	endogenous retrovirus group H, member 4	14q32.2	clone c4.2	U35031, U88895	[50]

ERVH-5	endogenous retrovirus group H, member 5	10p12.1	clone c14.6	U35033	[50]

ERVH-6	endogenous retrovirus group H, member 6	Yq11.223		U88898	[50]

ERVH-7	endogenous retrovirus group H, member 7	14q32.12		BC039675, T67812	[50]

ERV9-1	endogenous retrovirus group 9, member 1	11q13.2	pTR2	X15673, X15675, X57147	[51] [52]

^a^The ERVK group is referred to as HERV-K(HML-2) according to a nomenclature introduced by Andersson *et al*. in 1999 [53]. ^b^The ERVK3 group has been referred to as HERV-K(HML-6) according to the Andersson *et al*. classification scheme [53]. ^c^The following loci have not appeared in publications but have been annotated by the RefSeq project: ERVFRD-2 (Entrez Gene:388560); ERVI-1 (Entrez Gene ID:100131068); ERV18-1 (Entrez Gene ID:100133791); ERVMER61-1 (Entrez Gene ID:339476).

## Previous ERV nomenclature and the Human Genome Organisation Gene Nomenclature Committee

The Human Genome Organisation (HUGO) Gene Nomenclature Committee (HGNC) works under the auspices of HUGO and is the only worldwide authority that assigns standardised nomenclature to human genes [17]. The HGNC has previously focused on approving nomenclature for protein-coding genes, pseudogenes, phenotypes and noncoding RNA. In the past, the committee has approved symbols for specific human ERVs only at the request of individual researchers. The symbols did not follow a systematic nomenclature: some symbols were of a simple format (for example, ERV1), some provided information on the group to which the ERV belonged (for example, ERVK2) and others included information on proteins encoded by the ERV (for example, ERVWE1 (endogenous retroviral family W, *env*(C7), member 1)). On reviewing the literature, it was clear that (1) many of the most frequently published loci were not represented by HGNC symbols, (2) by following more than one system, HGNC symbols were not serving the community, and (3) the nomenclature needed both updating and expansion.

HGNC editors curate relevant information for each gene that has approved nomenclature. In addition to approving a gene symbol and name for each transcribed human ERV, the HGNC records all known symbol aliases so that information on each gene can be retrieved using any known symbol. HGNC entries also include the chromosomal location of the ERV locus, links to GenBank, European Molecular Biology Laboratory (EMBL) and DNA Databank of Japan (DDBJ) sequence records and links to at least one PubMed reference. Where appropriate, links are also provided to annotation projects at both the genomic and proteomic levels. HGNC names are propagated to other major biological databases, such as Ensembl, UniProt and Entrez Gene. Therefore, this new nomenclature will provide a useful resource that is currently unavailable to the ERV community and other researchers concerned with ERVs.

## A gene-based nomenclature

The primary definition of a gene used by the HGNC is "a DNA segment that contributes to phenotype/function" [18]. It is beyond the scope of this nomenclature effort to standardise the nomenclature of ERVs in general or to attempt to name every ERV element in the genome. As discussed above, there is evidence that some human ERVs encode functional proteins and that some encode transcripts and/or proteins which may be associated with disease, so the transcriptionally active loci come under the remit of the HGNC for naming. This category of ERVs represents most of the individual loci that have been published with individual names, so it is worth developing a standardised nomenclature for this subset. The three criteria for being accepted as a transcriptionally active ERV are as follows: (1) The ERV must be represented by an mRNA sequence in a public database, (2) the reported cDNA sequence must map unambiguously to the reference genome to allow identification and (3) the sequence must represent a viral gene rather than solely a solitary LTR. We acknowledge that there are sources of uncertainty. Many ERVs may be expressed at a low level [19], a "leakage" which can be hard to distinguish from perhaps more significant expression. Groups of recently integrated ERVs may be highly expressed, but their transcripts may be identical or almost identical and could be hard to map unambiguously. However, these difficulties should not prevent the naming of ERV loci which fulfil the criteria mentioned above. There is one symbol approved per ERV locus independently of how many viral genes the ERV may encode.

## A systematic ERV nomenclature scheme

The nomenclature scheme described in this paper aims to be concise so that it is user-friendly. It also aims to be informative to researchers, including those who are less familiar with the field. To be informative, the nomenclature scheme is hierarchical, with each symbol beginning with the root symbol "ERV" so that the symbols are instantly recognisable and can be grouped together in searches. Note that many researchers have published papers using symbols beginning with "HERV", but it is against the guidelines of the HGNC ever to use H for "human" in symbols, mainly because this precludes the possibility of the nomenclature scheme's being extended to other species. Each ERV symbol, then, includes an identifier that represents the group to which the ERV belongs.

In order for the nomenclature scheme to be systematic, one method of sorting ERVs into groups needed to be selected. The Repbase system [6] is a widely known, comprehensive database of repetitive elements that groups ERVs together on the basis of sequence similarity. RepeatMasker annotations using Repbase designations are available on the University of California, Santa Cruz (UCSC) [20], and Ensembl [21] genome browsers, making these ERV groups highly accessible and recognisable to researchers in the field. Therefore, the nomenclature system uses the Repbase classification system for naming the ERVs within groups. Repbase groups, however, do not follow a systematic nomenclature and often contain an unallowable "H" for "human". When deciding on the group identifier to be included in each symbol, we compared Repbase symbols with those that have appeared frequently in the literature. In cases where there was a well-supported nomenclature present in the literature, we used this symbol in place of the Repbase symbol; for example, we used ERVW instead of the Repbase group designation HERV17, as we felt that these would be more likely to be used by the ERV community. For a comparison of the group symbols used in the new nomenclature scheme with Repbase designations, see Table 2.

**Table 2:** Comparison of Repbase group symbols with group symbols used in the nomenclature scheme presented herein

Repbase group symbol	Group symbol in new nomenclature scheme
HERVK	ERVK
HERVK3	ERVK3
MER50	ERVFRD
ERV3	ERV3
PABL_B	ERVPABLB
HERV-Fc1	ERVFC1
HERV17	ERVW
HERVS71	ERVS71
HERVH48	ERVH48
HERVFH21	ERVFH21
HERVE	ERVE
HERV18	ERV18
MER61	ERVMER61
HERVH	ERVH
HERV9	ERV9

Finally, each ERV within a particular group is uniquely identified by a number, for example, ERVK-1. Numbers are assigned consecutively within each group to make the nomenclature system expandable. The number is used to make each symbol unique and has no intrinsic meaning. ERVK-2 has merely been assigned the next number following ERVK-1, but this provides no information on the position of the ERVs within the genome or the order in which an ERV may have been published. The use of numerical identifiers keeps the symbols as short as possible to encourage widespread use by researchers. Newly identified transcribed loci will take the next available consecutive number for their particular group; for example, if a newly transcribed ERVK locus is identified, it will take the symbol ERVK-26. Each symbol is accompanied by an expanded gene name which clearly and succinctly explains that derivation of the nomenclature; for example, the full name of ERVFRD-1 is "endogenous retrovirus group FRD, member 1".

We are aware that the proposed nomenclature scheme cannot encompass all conceivable (and sometimes known) unusual structures of ERV loci, such as hybrid loci consisting of different ERV groups and ERV insertions into existing ERV loci [22]. HGNC, after conferring with researchers who submit newly identified transcribed loci, will decide whether or how to name such unique loci on a case-by-case basis. For example, the scheme will not incorporate ERV locus transcripts that are part of another gene's transcript, as these elements will not be considered separate loci.

Table 1 lists transcribed human ERVs that have been named according to the new nomenclature system. All ERVs in the table either have been published or have been annotated by the RefSeq project. An initial list was sent to a number of researchers in the field for their comments. The list was expanded as these researchers suggested more loci. Where no transcript sequence was available, authors were asked to submit representative sequences to the GenBank, EMBL and DDBJ databases. We encourage researchers to contact the HGNC if they know of further ERVs that can be included in the scheme.

Finally, although only human gene nomenclature is under the remit of the HGNC, we wish to mention that the naming system introduced here for transcribed ERVs could, in principle, also be applied to other, non-ERV repetitive sequences in the human genome, as well as to repetitive DNA in nonhuman species. Future research will probably reveal numerous transcribed repetitive DNA sequences in various species. Judged just from ERV designations in different species, a standardised naming system for transcribed repeat loci may be highly beneficial to avoid future confusion.

## Competing interests

The authors declare that they have no competing interests.

## Authors' contributions

All authors contributed to the presented nomenclature scheme and wrote the manuscript.
